# Purification and Structural Analysis of the Effective Anti-TMV Compound ε-Poly-l-lysine Produced by *Streptomyces ahygroscopicus*

**DOI:** 10.3390/molecules24061156

**Published:** 2019-03-23

**Authors:** Jianguang Chen, He Liu, Zihao Xia, Xiuxiang Zhao, Yuanhua Wu, Mengnan An

**Affiliations:** College of Plant Protection, Shenyang Agricultural University, Shenyang 110866, Liaoning, China; chenjianguang2010@126.com (J.C.); m18240156631_2@163.com (H.L.); zihao8337@syau.edu.cn (Z.X.); zhaoxx0772@163.com (X.Z.)

**Keywords:** *Streptomyces ahygroscopicus*, anti-TMV activity, isolation and purification, structural determination, ε-poly-l-lysine

## Abstract

Microbial secondary metabolites produced by actinomycetes are important natural products widely applied to control plant diseases. A variety of actinomycetes were isolated from soil samples collected from Tianzhu Mountain in Shenyang, China. A *Streptomyces* strain Shenyang Tianzhu (STZ) exhibits effective antiviral activity against Tobacco mosaic virus (TMV). The isolate was identified as *Streptomyces ahygroscopicus* based on its cultural, morphological, physiological, biochemical characteristics as well as the phylogenetic analysis using 16S rRNA sequences. To obtain the pure anti-TMV compound from *Streptomyces* STZ, the culture broth was subjected to Amberlite IRC-50 ion-exchange resin, SX-8 macroporous adsorption resin and Sephadex G-25 gel column chromatography. The purified active compound was confirmed to be ε-poly-l-lysine (ε-PL), with molecular mass in the range of 3454–4352 Da by structural analysis with infrared (IR), matrix-assisted laser desorption ionization-time-of-flight MS (MALDI-TOF), thin-layer chromatography (TLC) and high-resolution magic angle spinning nuclear magnetic resonance (HR-MAS NMR). The protective and curative effects of the purified compound ε-PL were tested and the results showed that the compound exhibited significant protective and curative activity against TMV. The potential application of ε-PL as an efficient anti-plant virus agent was expected.

## 1. Introduction

Plant virus diseases cause considerable economic losses in cereal and horticulture production worldwide every year [1]. Tobacco mosaic virus (TMV) is capable of infecting more than 885 plant species in 65 families [2,3]. Chemical pesticides and insecticides play important roles in controlling virus disease, but the antiviral efficacy and effective duration of action are still limited in the fields. Therefore, development and commercialization of highly effective antiviral agents are expected.

Plant virus inhibitors from metabolites of microbes have been considered as a potential alternative for chemical pesticides, which have become an increasing research spotlight. These bio-agents have various advantages in the respect of low mammalian toxicity, biodegradability, good environmental compatibility and a unique mode of action [4]. Several bio-agents exhibit antiviral activity against TMV have been previously reported. Ningnanmycin is an effective anti-plant virus agent that was isolated from the fermentation broth of *Streptomyces noursei* var. *xichangensis*. It effectively inhibited assembly of virions by inhibiting the polymerization of TMV coat protein (CP) *in vitro* and systemically induced the accumulation of pathogenesis-related proteins (PRs) [5]. Cytosinpeptidemycin (CytPM) is a registered antibiotic for agriculture from Agriculture Ministry of China, which was produced by *Streptomyces ahygroscopicus* isolated from soil samples of Liaoning Province, China. It displays a broad antiviral activity against many plant viruses, such as TMV and rice stripe virus (RSV) [6,7,8]. Furthermore, CytPM has been used to control southern rice black-streaked dwarf virus (SRBSDV), and has been shown to induce stress and defense responses, such as expression of heat shock protein (Hsp) and pathogenesis-related protein 5 (PR-5) [8]. Tan et al. reported an exocellular polysaccharide (EP) from an endophytic fungus (*Phomopsis* sp FJBR-11) showed a significant inhibition against TMV at a half-maximal inhibitory concentration (IC50) of 1.08 µg/mL [9]. Additionally, studies have shown that a novel glycoprotein (GP-1) from *Streptomyces kanasensis* can induce systemic resistance (ISR) and exhibits extensive inhibitory effect on TMV infection [10]. However, barely any of the biological agents have been successfully registered as anti-viral agent and widely applied in the field. Therefore, discovering novel, effective, practical, and environmentally safe antiviral agents remains an urgent task to be developed. 

In the present study, *Streptomyces* strain STZwas isolated from the soil of Tianzhu Mountain in Liaoning Province, China, and showed significant anti-TMV activity. Here, we performed polyphasic taxonomic analysis of *Streptomyces* STZ. Moreover, a novel anti-TMV compound ε-poly-l-lysine (ε-PL) produced by the strain was purified by various chromatographic techniques, and its structure was determined by various spectral analysis. It is reported that ε-PL has been widely used as a natural food preservative, interferon inducer, drug delivery vehicle, and gene delivery vector, due to antibacterial activity, low toxicity, biodegradability, and thermal stability [11,12,13,14]. In this study, the potential application of ε-PL as an efficient anti-plant virus agent was proposed first time.

## 2. Results

### 2.1. Isolation, Morphological and Cultural Characteristics of Streptomyces STZ

**Figure 1:**
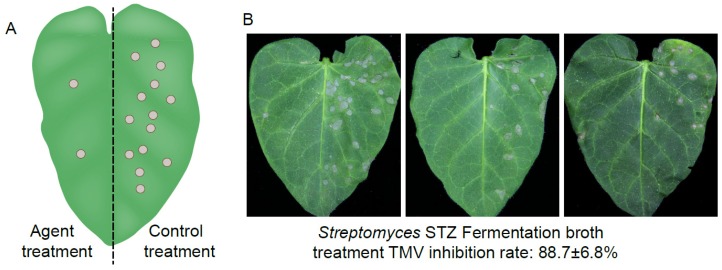
(**A**) Schematic representation of half-leaf method to study the anti-viral effect of the agent using *N. glutinosa* plants; (**B**) Inhibition effect of *Streptomyces* STZ fermentation broth against the TMV.

Total 69 actinomycete strains were isolated from 34 soil samples based on colony morphology and stability in subculture. Inhibitory activities of fermentation cultures against TMV using the isolated *Streptomyces* strains were assessed by the half-leaf method and the results indicated that fermentation of *Streptomyces* strain STZ exhibited the strongest anti-TMV effect with inhibition rate of 88.7 ± 6.8% (Figure 1). *Streptomyces* STZ showed excellent growth with abundant brownish to greyish brown aerial mycelia on ISP2 and Bennett media, showed moderate growth on ISP4 and ISP5 media with a clam white to light brown aerial mycelium after 14 days of incubation at 28 °C. No soluble diffusible pigments were observed on the tested media. The color of the substrate mycelium was light-brown on ISP2, yellow-gray on ISP4, and light mango brown on ISP5. 

### 2.2. Physiological and Biochemical Characteristics

The physiological and biochemical properties of *Streptomyces* STZ are shown in Table 1. Results revealed that it was able to reduce nitrate and hydrolyze starch, gelatin and cellulose but that hydrogen sulfide was not produced. The optimal growth temperature was 28 °C, and growth was inhibited at 50 °C. Strain STZ showed high tolerance to NaCl including good growth on medium supplemented with NaCl up to 5%, poor growth at 7%, and no growth at 10%. Utilization of various carbon sources by the isolate indicated that it could grow on d-glucose, sucrose, starch, l-arabinose, d-fructose, galactose, d-mannitol, d-Xylose, inositol and maltose but not l-rhamnose and raffinose.

### 2.3. 16S rRNA Sequence Analysis

**Figure 2:**
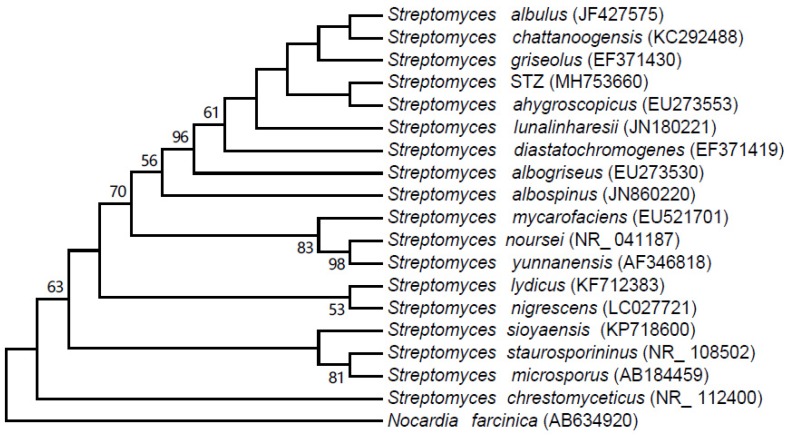
Phylogenetic analysis based on 16S rRNA gene sequences of *Streptomyces* STZ and species of the genus *Streptomyces* using the maximum likelihood method. Numbers on branch nodes are bootstrap values (1000 replicates).

A partial 16S rRNA gene sequence (1435 nucleotides) of strain STZ was determined and deposited with accession number MH753660 in the GenBank database. Comparative 16S rRNA gene sequence analysis using BLAST showed that the strain could be classified as a member of the genus *Streptomyces* and shared high sequence identity (99.37%) with *Streptomyces ahygroscopicus*. A 16S rRNA gene-based phylogenetic tree was constructed with the maximum likelihood method with the different *Streptomyces* reference species available in the GenBank database (Figure 2). Phylogenetic analysis indicated that strain STZ closely clustered with the strain *Streptomyces ahygroscopicus* (accession number EU273553).

### 2.4. Purification and Structure Elucidation of the Pure Compound 

**Figure 3:**
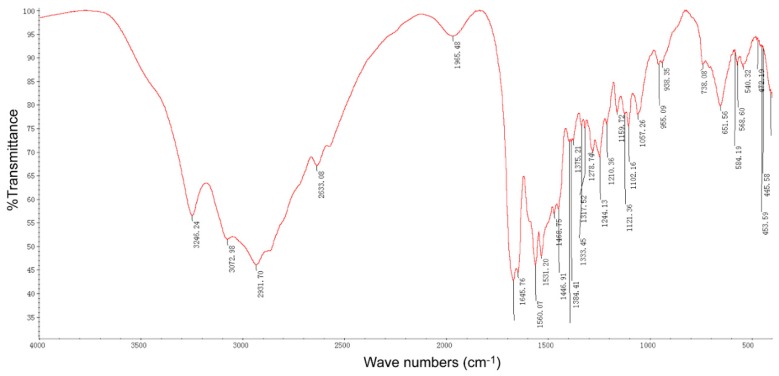
IR spectrum of the pure active compound isolated from *Streptomyces* STZ.

A pure compound was obtained from the fermentation broth of strain STZ after separation and purification procedures, the compound with faint yellow powder and slight bitter taste. The IR spectrum of the compound showed strong fluorescent (Figure 3). In the IR spectrum, amino group stretching vibration was observed at 3246 cm^−1^, alkyl group stretching vibration at 2931–3072 cm^−1^, carbonyl group stretching vibration at 1668 cm^−1^; peaks at 1560 and 651cm^−1^ were assigned to in-plane and out-of-plane bending vibration absorption of amino groups, respectively. Intriguingly, the spectrum was generally consistent with the previously reported IR spectrum of ε-PL [15].

**Figure 4:**
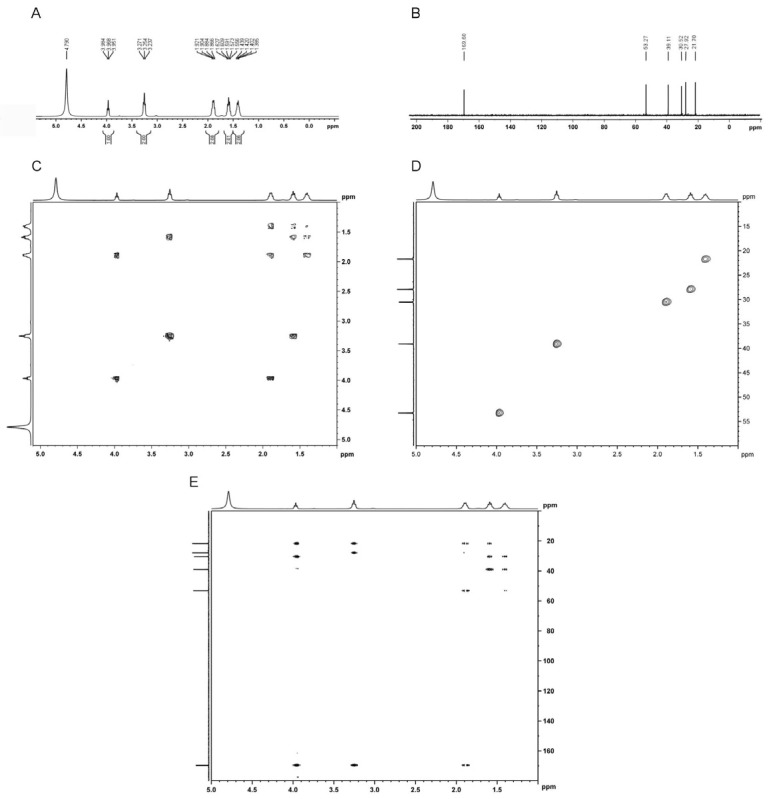
(**A**) ^1^H-NMR spectrum of the pure active compound produced by *Streptomyces* STZ; (**B**) ^13^C-NMR spectrum of the pure active compound produced by *Streptomyces* STZ; (**C**) H-HCOSY spectrum of the pure active compound produced by *Streptomyces* STZ; (**D**) HSQC spectrum of the pure active compound produced by *Streptomyces* STZ; (**E**) HMBC spectrum of the pure active compound produced by *Streptomyces* STZ.

The ^1^H-NMR spectrum showed nine H signals in the higher field (<6 ppm), which were attributable to the chemical shift of protons on the saturated sp3 carbon atom (Figure 4A). ^13^C-NMR and DEPT spectra showed six carbon signals (Figure 4B), of which there were one quaternary carbon, one tertiary carbon and four secondary carbon carbons. H-HCOSY experiment showed δ1.385–1.439 (C4-H) was correlated with δ1.866–1.921 (C3-H) and δ1.556–1.627 (C5-H); δ1.556–1.627 (C5-H) was correlated with δ1.385–1.439 (C4-H) and δ3.237–3.271 (C6-H); δ1.866–1.921 (C3-H) was correlated with δ1.385–1.439 (C4-H) and δ3.951–3.984 (C2-H); δ3.237–3.271 (C6-H) was correlated with δ1.556–1.627 (C5-H); δ3.951–3.984 (C2-H) was correlated with δ1.866–1.921 (C3-H) (Figure 4C). HSQC spectrum showed δ21.70 (C4) had a correlation with δ1.385–1.439 (C4-H); δ27.92 (C5) had a correlation with δ1.556-1.627 (C5-H); δ30.52 (C3) had a correlation with δ1.866-1.921 (C3-H); δ39.11 (C6) had a correlation with δ3.237-3.271 (C6-H); and δ53.27 (C2) had a correlation with δ3.951–3.984 (C2-H) (Figure 4D). Results of HMBC spectrum indicated that δ21.70 (C4) long-range correlations with δ3.951–3.984 (C2-H), δ3.237–3.271 (C6-H), δ1.866–1.921 (C3-H), and δ1.556–1.627 (C5-H); δ27.92 (C5) long-range correlation with δ3.237–3.271 (C6-H); δ30.52 (C3) long-range correlations with δ3.951–3.984 (C2-H), δ1.385–1.439 (C4-H), and δ1.556–1.627 (C5-H); δ39.11 (C6) long-range correlations with δ1.385–1.439 (C4-H) and δ1.556–1.627 (C5-H); δ53.27 (C2) long-range correlation with δ1.866–1.921 (C3-H); δ169.60 (C1) long-range correlations with δ3.951–3.984 (C2-H), δ3.237–3.271 (C6-H) and δ1.866–1.921 (C3-H) (Figure 4E).

The hydrolytes were analyzed by TLC with ε-PL and l-lysine standards used as controls. Results indicated that only lysine was present as an amino acid in the hydrolyte, which suggested that the purified compound produced by strain STZ was a polymer polymerized by l-lysine. Finally, the product was confirmed to be ε-PL that was in consistent with results from previous reports [16,17].

### 2.5. MALDI-TOF-MS Analysis of the Active Compound 

**Figure 5:**
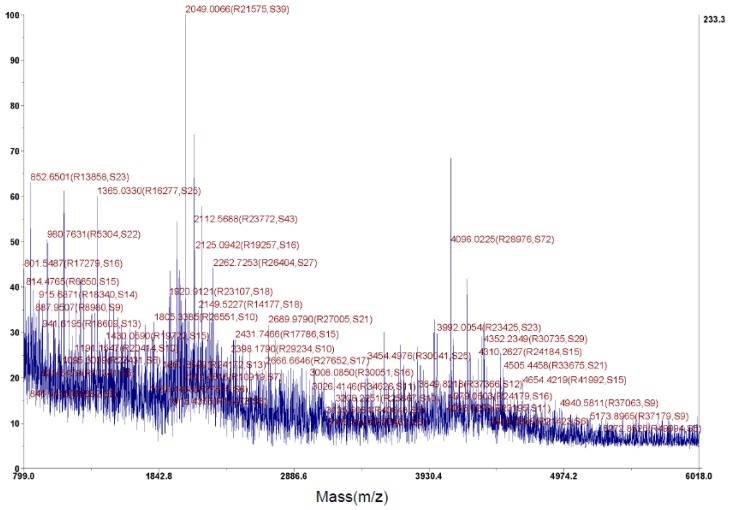
MALDI-TOF MS spectrum of the purified active product from *Streptomyces* STZ.

As ε-PL belongs to homo-poly-amino acid with a chain of lysine residues (molecular weight: 146.19) linked by dehydration of water (molecular weight: 18.02), its molecular weight was calculable by the formula 146.19 × n − 18.02 × (n − 1), where n is the number of lysine residues. The relative molecular mass of the active compound was estimated by MALDI-TOF-MS (Figure 5). The results indicated a series of [M+H]^+^ values that fit the estimated formula. The molecular mass was distributed in the range of 3454–4352 Da; the *m*/*z* 4,096 represent the main molecular ion peak, and the *m*/*z* 2049 represent the double-charged ion peak. The polymerization degree was deduced to be in the range of 27–34.

### 2.6. Protective and Curative Activities of the Compound ε-PL Against TMV

The protective and curative effects of the purified compound ε-PL against TMV were tested at two concentrations using the half-leaf method. The results showed that compound ε-PL exhibited significant protective activity (71.4 ± 1.7% at 500 μg/mL and 84.2 ± 3.2% at 1000 μg/mL) and curative activity (59.3 ± 2.9% at 500 μg/mL and 68.7 ± 3.4% at 1000 μg/mL, respectively) against TMV (Table 2).

## 3. Discussion

The identification of easily biodegradable and ecofriendly compounds to meet the ongoing demand for novel antiviral agents, especially those produced by *Streptomyces* species, has increased in recent years.

In this study, the morphological, physiological, and biochemical properties and the phylogenetic evaluation suggest that the *Streptomyces* strain STZ belonged to *Streptomyces ahygroscopicus*. Finally, a pure active compound was isolated from the fermentation broth by various chromatographic techniques. Based on the analysis including TLC, UV spectroscopy, IR spectroscopy, HR-MAS-NMR spectroscopy and MALDI-TOF-MS, the compound was confirmed to be ε-PL, with molecular mass in the range of 3454–4352 Da and a degree of polymerization between 27–34.

ε-PL is an unusual biopolymer composed of l-lysine residues connected between α-carboxyl and ε-amino groups, generally comprising 25–30 l-lysine residues [18]. ε-PL has previously been identified as positive substance in the culture supernatant of *Streptomyces albulus* from Dragendorff’s reagent [18]. To date, increasing numbers of ε-PL producing microbes, such as *Kitasatospora kifunense*, *Epichloe* sp., *Streptomyces aureofaciens*, *Streptomyces griseoaurantiacus*, *Streptomyces roseoverticillatus*, *Streptomyces diastatochromogenes,* and *Bacillus* sp. have been reported [19,20,21]. 

ε-PL has been found to effectively inhibit broad ranges of microorganisms, including gram-positive and gram-negative bacteria such as *Escherichia coli*, *Bacillus subtilis*, *Lactobacillus*, *Staphylococcus aureus*, *Botrytis cinerea*, yeasts and certain types of viruses and phages [22,23,24,25]. In addition, ε-PL also has anticancer activity, which can inhibit the proliferation of the Hela S3 cells with a lethal concentration of LC71 and HepG2 cells with a lethal concentration of LC53, respectively [26]. Moreover, ε-PL is edible, water-soluble, biodegradable, and non-toxic toward to humans and the environment [27,28]. Due to the positive attributes described above, ε-PL has been approved by regulatory agencies of the United States, Japan, Korea, and Europe as a preservative in food manufacturing [29]. In addition, ε-PL has been specifically used as an emulsifying agent, a gene and drug carrier, an endotoxin-selective removal additive, and a cosmetic agent [30,31,32].

However, there have been no relevant reports on ε-PL as an anti-virus agent in agricultural applications. In this study, ε-PL produced by the *Streptomyces ahygroscopicus* was evaluated for the first time for its antiviral effects against plant virus. Whereas the precise molecular anti-viral mechanisms remained to be clarified by the future works. The present work also indicats there is potential for significant commercial applications of the compound ε-PL in the control of plant virus diseases.

## 4. Materials and Methods

### 4.1. Sample Collection and Isolation of Streptomyces Strains

A total of 34 soil samples were collected from Tianzhu Mountain in Shenyang, Liaoning Province, China (41°48′N, 123°25′48′′E). The soil samples were air dried for 7 days at room temperature. *Streptomyces* strains were isolated by the standard dilution plate method on Gause’s synthetic agar medium. 2.5 mL of K_2_Cr_2_O_7_ solution (1.775 g/L) was added to 100 mL medium as antifungal agent [33]. The cultures were incubated at 28 °C for 21 days. Single colonies were selected and purified 5 times. The purified cultures were maintained on slopes at 4 °C and suspended in a sterile glycerol solution 25% (*w*/*v*) at −80 °C for further study. 

### 4.2. Fermentation

The composition of the seed and fermentation medium was as follows: 50 g/L glucose, 10 g/L (NH4)_2_SO_4_, 5 g/L yeast extract, 0.5 g/L MgSO_4_·7H_2_O, 1.4 g/L K_2_HPO_4_, 0.04 g/L ZnSO_4_·7H_2_O and 0.03 g/L FeSO_4_·7H_2_O, and pH 6.8. The test strains were inoculated on the ISP3 medium and incubated at 28 °C for 7 days. Two spore cakes (5 mm in diameter) were removed and inoculated into a 250 mL flask containing 50 mL of seed medium. The seed cultures were grown at 28 °C at an agitation speed of 180 rpm for 24 h. Subsequently, seed cultures were inoculated aseptically into 250 mL flasks containing 50 mL of fermentation medium at a final concentration of 6%. The fermentation was carried out on a rotary shaker (Shanghai Zhichu Instruments, Shanghai, China) at an agitation speed of 180 rpm at 28 °C for 72 h. Biomass was separated from the growth medium by centrifugation at 8000 rpm for 15 min. The supernate was stored at 4 °C for subsequent use.

### 4.3. Anti-TMV Activity Screening of Streptomyces Strains

*Nicotiana glutinosa* at the 6–8 leaf stage were used as local lesion exhibiting host plants inoculated with TMV, which was maintained on *Nicotiana tabacum* cv. K326. TMV virions were purified according to Gooding’s method [34]. The purified virus was diluted to 30 µg/mL with 0.01 M phosphate-buffered saline (PBS) before use. Test of anti-TMV activity was carried out by the half-leaf method as described [35]. The culture filtrate was diluted five times and equally mixed with TMV (30 µg/mL). After 30 min, the mixture was mechanically inoculated onto the left side of *N. glutinosa* leaves and a mixture of distilled water and TMV was inoculated onto the right side of the same leaf as a control. Each inoculated leaf was washed with water after 10 min. *N. glutinosa* were cultivated at 25 °C. The numbers of local lesions were recorded 3–4 days after inoculation. Three replicates were conducted to ensure the reliability of the results. The inhibitory rate was calculated according to the following formula: Inhibition rate (%) = [(C − T)/C] × 100%. Where T is the average number of local lesions with culture filtrate treatment and C is the average number of local lesions of the control.

### 4.4. Taxonomic Characterization of the Strain STZ

Culture features were investigated on various international *Streptomyces* Project (ISP) media and Bennett’s agar [36,37]. The color of mycelia, the degree of growth and other features were recorded following incubation at 28 °C for 14 days. Analyses for the production of H_2_S liquefaction of gelatin, reduction of nitrate, hydrolysis of starch, cellulose and utilization of carbon, nitrate reduction reaction, salt tolerance, and ability to grow at different temperatures were carried out as previously described [38,39,40].

Genomic DNA from strain STZ was extracted using the method as described [41]. Partial sequence of the 16S rRNA gene was amplified by PCR using the universal primers 27F (5′-AGAGTTTGATCCTGGCTCAG-3′) and 1492R (5′-TACCTTGTTACGACTT-3′) [42]. The PCR reaction was carried out with an initial denaturation at 94 °C for 4 min followed by 30 cycles of denaturation at 94 °C for 1 min, annealing at 55 °C for 1 min, and extension at 72 °C for 2 min. After the cycling, a final extension at 72 °C for 7 min was performed. The PCR product was purified and integrated into the vector pMD19-T (Takara, Japan) and the 16S rRNA gene was sequenced by Sangon Biotech Co., Ltd. (Shanghai, China). 

The sequence data was deposited in GenBank (no. MH753660) and compared with sequences using the nucleotide BLAST (http://www.ncbi.nlm.nih.gov/BLAST) in GenBank. Multiple alignments of the sequence and the closest matches retrieved from the database and the phylogenetic tree were constructed with Mega program version 7.0.14 using the maximum likelihood method [43].

### 4.5. Separation and Purification 

For isolation of the anti-TMV compound from the culture of strain STZ, bioassay based on anti-TMV activity was performed using the half-leaf method in every process during purification. Method to assess the purification of the active compound was as below: fermentation broth was centrifuged at 8000 rpm for 15 min to remove mycelium, the supernate was adjusted to pH 8.5 with 2 M NaOH and subjected to heating treatment at 65 °C for 1 h. Thereafter, the solution was filtered through filter paper to remove the precipitation. The filtrate was adsorbed onto an ion exchange column filled with Amberlite IRC-50 resin, deionized water was employed for washing resin, and 0.1 M HCl was used to elute active compound from resin. Subsequently, the pigment was removed by column chromatography on SX-8 macroporous adsorption resin. The active fractions were concentrated under vacuum and further purified by column chromatography using Sephadex G-25. Finally, the pure product was obtained by precipitation with acetone.

### 4.6. Structure Determination for the Pure Compound

The infrared (IR) spectroscopy spectrum was recorded using a KBr disk on a Nicolet 6700 Fourier infrared spectrometer (Thermo Fisher Scientific, Waltham, MA, USA). Proton NMR (^1^H-NMR) and a carbon NMR (^13^C-NMR) spectra of the compound were ascertained via AVANCE DRX-400 MHz NMR spectroscopy (Bruker, Rheinstetten, Germany). Additional heteronuclear multiple bond correlation (HMBC) spectroscopy, H-H correlation spectroscopy (H-H COSY) and heteronuclear singular quantum correlation (HSQC) analyses were also performed for structure elucidation. The amino acid composition of the pure compound was analyzed via hydrochloric acid hydrolysis and thin-layer chromatography (TLC). A sample (1 mL) of the pure product was hydrolyzed completely by heating in 6 mol/L HCl at 110 °C for 20 h. The reaction product was analyzed by TLC (developer, n-butanol-acetic acid-water-methanol at 3:1:2:2 relative amounts; detection, 0.5% ninhydrin reagent in ethanol) with ε-PL and lysine standards as controls.

### 4.7. Molecular Mass Determination of the Pure Compound

Mass spectrometry (MS) of the pure product was determined by matrix-assisted laser desorption ionization-time-of-flight mass spectrometry MS (MALDI-TOF-MS) using an AB TOF 5800 model (AB Sciex, Chicago, IL, USA). As a matrix, α-cyano-4-hydroxycinnamic acid was used (CHCA).

### 4.8. Protection Effect of the Compound Against TMV In Vivo 

The purified compound was solubilized in PBS and sprayed on the left side leaves with 6–8 leaf stage old *N. glutinosa*, then PBS buffer was used as a control on the right side of leaves. The leaves were then inoculated by the 100 μL virions of TMV at concentration of 30 ng/μL after 12 h. The local lesions and inhibition rates were calculated at 3–4 days. Each assays were repeated for at least 3 times.

### 4.9. Curative Effect of the Compound Against TMV In Vivo 

The leaves on *N. glutinosa* of the same age were selected and inoculated with the 100 μL 30 ng/μL TMV virions. After 12 h, the compound solution was sprayed on the left side and the PBS buffer was sprayed on the right side for control. The local lesion numbers were then recorded 3–4 days after inoculation. 

## Figures and Tables

**Table 1:** Physiological and biochemical characteristics of the strain STZ.

Tests	Result	Tests	Result
**Biochemical tests:**		**Carbon source utilization:**	
Cellulose decomposition	−	d-glucose	+
Starch hydrolysis	+	Sucrose	+
Gelatin hydrolysis	+	l-arabinose	+
Nitrate reduction	+	Starch	+
H_2_S production	−	Raffinose	−
**NaCl tolerance (%, *w/v*):**		d-fructose	+
5	+	Maltose	+
7	+	d-Xylose	+
10	−	Galactose	+
**Growth temperature (°C):**		d-mannitol	+
20	+	Inositol	+
28	+	l-rhamnose	−
37	+		
50	−		

+ positive; − negative.

**Table 2:** Protection and curation effect of active compound against TMV.

Compound Treatment	Protective Effect	Curative Effect
500 μg/mL	71.4 ± 1.7%	59.3 ± 2.9%
1000 μg/mL	84.2 ± 3.2%	68.7 ± 3.4%

## References

[1] Scholthof K.G., Adkins S., Czosnek H., Palukaitis P., Jacquot E., Hohn T., Hohn B., Saunders K., Candresse T., Ahlquist P. (2011). Top 10 plant viruses in molecular plant pathology. Mol. Plant Pathol..

[2] Fraile A., Garcia-Arenal F. (2018). Tobamoviruses as Models for the Study of Virus Evolution. Adv. Virus Res..

[3] Roossinck M.J. (2015). Plants, viruses and the environment: Ecology and mutualism. Virology.

[4] Seiber J.N. (2011). Sustainability and agricultural and food chemistry. J. Agric. Food Chem..

[5] Han Y.G., Luo Y., Qin S.R., Xi L., Wan B., Du L.F. (2014). Induction of systemic resistance against tobacco mosaic virus by Ningnanmycin in tobacco. Pestic. Biochem. Physiol..

[6] Zhao X.X., Wu Y.H., Du C.M., Song Y. (2004). Control of tomato virus disease with Cytosintetidemycin. Pesticides.

[7] Zhu C.Y., Wu Y.H., Wang C.M., Zhao X.X., Wang Y.H., Du C.M. (2005). Inhibition of Cytosinpeptidemycin on tobacco mosaic virus. Plant Prot..

[8] Yu L., Wang W.L., Zeng S., Chen Z., Yang A.M., Shi J., Zhao X.Z., Song B.A. (2018). Label-free quantitative proteomics analysis of Cytosinpeptidemycin responses in southern rice black-streaked dwarf virus-infected rice. Pestic. Biochem. Physiol..

[9] Tan Q.W., Fang P.H., Ni J.C., Gao F.L., Chen Q.J. (2017). Metabolites Produced by an Endophytic *Phomopsis* sp. and their anti-TMV activity. Molecules.

[10] Han L.R., Zhang G.Q., Miao G.P., Zhang X., Feng J.T. (2015). *Streptomyces kanasensis* sp. nov., an antiviral glycoprotein producing *Streptomyces* isolated from forest soil around Kanas Lake of China. Curr. Microbiol..

[11] Shima S., Matsuoka H., Iwamoto T., Sakai H. (1984). Antimicrobial action of epsilon-poly-l-lysine. J. Antibiot..

[12] Neda K., Sakurai T., Takahashi M., Ashiuchi M., Ohgushi M. (1999). Two-generation reproduction study with teratology test of ε-poly-l-lysine by dietary administration in rats. Jpn. Pharmacol. Ther..

[13] Hiraki J. (1995). Basic and applied studies on ε-poly-l-lysine. J. Antibact. Antifung. Agents.

[14] Hamano Y. (2011). Occurrence, biosynthesis, biodegradation, and industrial and medical applications of a naturally occurring epsilon-poly-l-lysine. Biosci. Biotechnol. Biochem..

[15] Shima S., Sakai H. (1981). Poly-l-lysine Produced by *Streptomyces*. Part III. Chemical Studies. Agric. Biol. Chem. Chem..

[16] Maeda S., Kunimoto K.K., Sasaki C., Kuwae A., Hanaic K. (2003). Characterization of microbial poly (ε-l-lysine) by FT-IR, Raman and solid state ^13^C-NMR spectroscopies. J. Mol. Struct..

[17] Maeda S., Mori T., Sasaki C., Kunimoto K., Kuwae A., Hanai K. (2005). Structural investigation of microbial poly (ε-l-lysine) derivatives with azo dyes by solid-state ^13^C and ^15^N NMR. Polym. Bull..

[18] Shima S., Sakai H. (1977). Polylysine produced by *Streptomyces*. Agric. Biol. Chem..

[19] Nishikawa M., Kobayashi K. (2009). *Streptomyces roseoverticillatus* produces two different poly(amino acid)s: Lariat-shaped gamma-poly (l-glutamic acid) and epsilon-poly(l-lysine). Microbiology.

[20] Takehara M., Hibino A., Saimura M., Hirohara H. (2010). High-yield production of short chain length poly (ε-l-ly sine) consisting of 5–20 residues by *Streptomyces aureofaciens*, and its antimicrobial activity. Biotechnol. Lett..

[21] Li S., Tang L., Chen X.S., Liao L.J., Li F., Mao Z.G. (2011). Isolation and characterization of a novel ε-poly-l-lysine producing strain: *Streptomyces griseofuscus*. J. Ind. Microbiol. Biotechnol..

[22] Li Y.Q., Han Q., Feng J.L., Tian W.L., Mo H.Z. (2014). Antibacterial characteristics and mechanisms of ε-poly-lysine against Escherichia coli and *Staphylococcus aureus*. Food Control.

[23] Li H., He C., Li G.G., Zhang Z.Q., Li B.Q., Tian S.P. (2019). The modes of action of epsilon-polylysine (ε-PL) against *Botrytis cinerea* in jujube fruit. Postharvest Biol. Technol..

[24] Ye R.S., Xu H.Y., Wan C.X., Peng S.S., Wang L.J., Xu H., Aguilar Z., Xiong Y.H., Zeng Z.L., Wei H. (2013). Antibacterial activity and mechanism of action of e-poly-l-lysine. Biochem. Biophys. Res. Commun..

[25] Shima S., Fukuhara Y., Sakai H. (1982). Inactivation of bacteriophages by ε-poly-l-lysine produced by *Streptomyces*. Agric. Biol. Chem..

[26] El-Sersy N.A., Abdelwahab A.E., Abouelkhiir S.S., Abou-Zeid D.M., Sabry S. (2012). Antibacterial and anticancer activity of ε-poly-l-lysine (ε-PL) produced by a marine *Bacillus subtilis* sp.. J. Basic Microbiol..

[27] Hiraki J. (2000). ε-Polylysine: Its development and utilization. Fine Chem..

[28] Hiraki J., Ichikawa T., Ninomiya S., Seki H., Uohama K., Seki H., Kimura S., Yanagimoto Y., Barnett J.W. (2003). Use of ADME studies to confirm the safety of ε-polylysine as a preservative in food. Regul. Toxicol. Pharmacol..

[29] Yamanaka K., Hamano Y., Hamano Y. (2010). Biotechnological production of poly-epsilon-l-lysine for food and medical applications. Amino-Acid Homopolymers Occurring in Nature.

[30] Shih I.L., Van Y.T., Shen M.H. (2004). Biomedical applications of chemically and microbiologically synthesized poly (glutamicacid) and poly(lysine). Mini-Rev. Med. Chem..

[31] Shih I.L., Shen M.H., Van Y.T. (2006). Microbial synthesis of poly (ε-lysine) and its various applications. Bioresour. Technol..

[32] Shukla S.C., Singh A., Pandey A.K., Mishra A. (2012). Review on production and medical applications of ε-polylysine. Biochem. Eng. J..

[33] Tsao P.H., Leben C., Keitt G.W. (1960). An enrichment method for isolating *Streptomyces* that produce diffusible antifungal antibiotics. Phytopathology.

[34] Gooding G.V., Hebert T.T. (1967). A simple technique for purification of tobacco mosaic virus in large quantities. Phytopathology.

[35] Song B.A., Zhang H.P., Wang H., Yang S., Jin L.H., Hu D.Y. (2005). Synthesis and antiviral activity of novel chiral cyanoacrylate derivatives. J. Agric. Food Chem..

[36] Shirling E.B., Gottlieb D. (1966). Methods for characterisation of *Streptomyces* species. Int. J. Syst. Bacteriol..

[37] Jones K.L. (1949). Fresh isolates of *Streptomyces* in which the presence of sporogenous aerial mycelia is a fluctuating characteristic. J. Bacteriol..

[38] Waksman S.A. (1950). The *Streptomyces*: Their nature, occurrence, activities, and importance. J. Am. Med. Assoc..

[39] Gottlieb D. (1961). An evolution of criteria and procedures used in the description and bcharacterization of *Streptomyces*. Appl. Microbiol..

[40] Holding A.J., Collee J.G. (1971). Chapter I routine biochemical tests. Methods Microbiol..

[41] Sharma A.D., Singh J. (2005). A nonenzymatic method to isolate genomic DNA from bacteria and *Streptomyces*. Anal. Biochem..

[42] Frank J.A., Reich C.I., Sharma S., Weisbaum J.S., Wilson B.A., Olsen G.J. (2008). Critical evaluation of two primers commonly used for amplification of bacterial 16S rRNA gene. Appl. Environ. Microbiol..

[43] Kumar S., Stecher G., Tamura K. (2016). MEGA7: Molecular Evolutionary Genetics Analysis Version 7.0 for Bigger Datasets. Mol. Biol. Evol..

